# Tell me y: anticipation of sex discrepancies in cell-free DNA testing due to maternal genetic abnormalities: a case report

**DOI:** 10.3389/fgene.2024.1502287

**Published:** 2025-01-20

**Authors:** Nuria Balaguer, Emilia Mateu-Brull, Jose Antonio Martínez-Conejero, Ana Cervero, Roser Navarro, Jorge Jiménez-Almazán, Miguel Milán

**Affiliations:** ^1^ Prenatal Diagnosis Department, Igenomix Spain Lab, Valencia, Spain; ^2^ Pre-implantation Genetic Testing for Monogenic Diseases Department, Igenomix Spain Lab, Valencia, Spain; ^3^ Bioinformatics Department, Igenomix Spain Lab, Valencia, Spain

**Keywords:** sex discordance, y-chromosome, maternal abnormalities, non-invasive prenatal testing (NIPT), cell-free (fetal) DNA, case-report

## Abstract

Sex discordance between cell-free DNA (cfDNA) testing and ultrasound examination is rare but can cause significant patient discomfort and uncertainty. Here, we present two clinical cases where a closer examination of raw sequencing data allowed us to anticipate possible discrepancies caused by the insertion of Y-chromosome regions into the maternal genome. We used Illumina’s VeriSeq NIPT Solution v2 and a proprietary bioinformatics pipeline to analyze cfDNA in the maternal bloodstream. Paired-end sequencing data were aligned to the reference genome (*hg19*). Non-duplicated aligned reads were aggregated into 100-kb bins, adjusted for CG bias, and further aggregated into 5-Mb windows. Z-scores were calculated for autosomes, sex chromosomes, and 5-Mb bins. The two clinical cases were classified as low-risk male fetuses according to the primary statistics (case A: NCV_x_ = 0.3; NCV_y_ = 40.6; native fetal fraction (FF_i_) = 5.1%, and case B: NCV_x_ = −0.3, NCV_y_ = 40.7, FF_i_ = 10.8%); however, the Y-chromosome-based FF (FF_y_) was significantly lower than the default FF estimate (FF_y_ ≅ 2% in both cases). Plots of X and Y chromosome Z-scores for each 5-Mb bin, according to genomic position, identified bins with Z-scores significantly higher than those expected for any pregnancy with a male fetus. The genomic coordinates of these bins overlapped with the amelogenin (*AMELY*) and protein kinase Y-linked (*PRKY*) genes, respectively. Amplification of these regions in the DNA isolated from the white blood cells fraction confirmed the presence of Y-chromosome insertions in the maternal genome. This study highlights a new source of discrepancy in cfDNA testing due to maternal genomic variations. These findings suggest the need for improvements to current bioinformatics pipelines to identify and exclude possible maternal perturbations from the classification algorithms used for aneuploidy and sex calls.

## Introduction

Cell-free DNA (cfDNA) testing has been established in many countries as the first-line screening method for major aneuploidies in early pregnancy, given the high level of sensitivity and specificity compared to traditional first-trimester biochemical screening (Zhang et al., 2015).

The ability of cfDNA testing to analyze sex chromosomes as early as 10 weeks of gestation has contributed to its popularity among parents desiring early information regarding fetal sex. A considerable number of patients prioritize fetal sex prediction over aneuploidy screening when deciding whether to undergo cfDNA testing (Stevens et al., 2023). Professional guidelines agree that patients undergoing cfDNA testing should receive pre- and post-test counseling, which would include a discussion of the possibility of false positives, sex discrepancies, incidental findings, and the variable expressivity and penetrance of the conditions screened for by the test, particularly sex chromosome aneuploidies and rare autosomal trisomies (Dungan et al., 2023; Wilson et al., 2013; Rose et al., 2022). This practice ensures the responsible application of cfDNA testing and enables evidence-based decision-making capacity for the prospective mother.

Discrepant results between cfDNA testing and ultrasound findings or other clinical information may originate from technical limitations and complex biological mechanisms occurring during the early stages of embryonic development. Sources of human error include blood sample mislabeling, laboratory methodological limitations, and suboptimal visualization of the external genitalia associated with limited ultrasound imaging performance at early gestational ages. Biological reasons for discordance include the presence of a vanishing twin, fetal-placental mosaicism for sex chromosome aneuploidies, a maternal transplant from a male donor, disorders of sexual development, or other fetal anomalies associated with abnormal or ambiguous external genitalia (Dhamankar et al., 2020).

Despite the numerous biological reasons already described, no scientific literature explicitly addresses cases where the insertion of Y chromosomal genetic material into the maternal genome represents the direct cause of a sex discrepancy in the context of cfDNA testing. This case report describes two instances where this specific mechanism was responsible for the observed discordance.

## Case report

### Patient 1 (case A)

**FIGURE 1:**
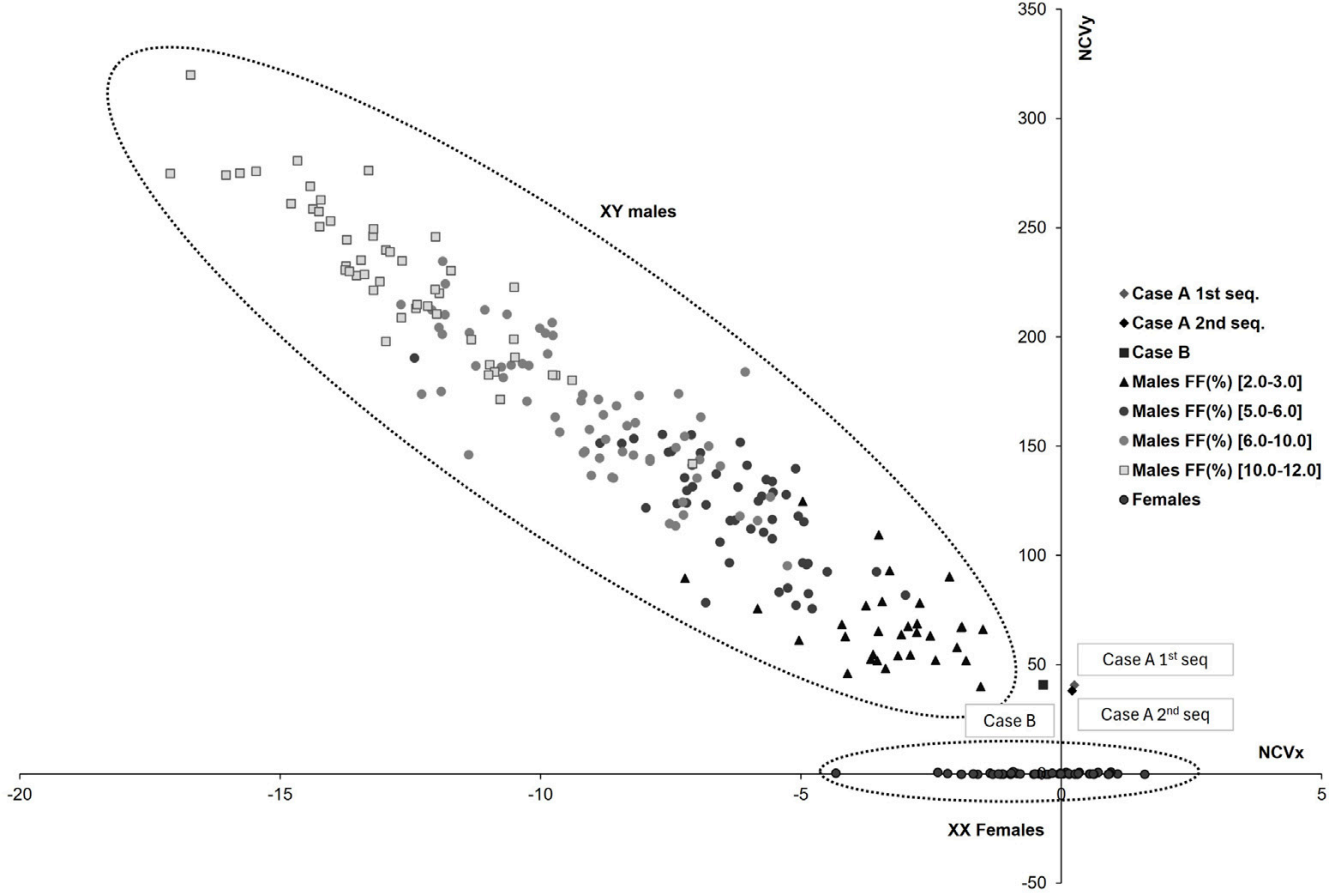
Sex chromosomes plot for different fetal fraction ranges. The horizontal axis represents the normalized chromosome value for the X chromosome (NCV_x_), and the vertical axis represents the normalized chromosome value for the Y chromosome (NCV_y_). The dotted lines indicate the normal range for female and male euploid samples across different fetal fraction (FF) ranges (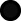) females with FFs between 2% and 12%; (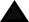) males with FFs between 2.0% and 3.0% (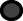) males with FFs between 5.0% and 6.0%; (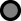) males with FFs between 6.0% and 10.0%; (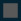) males with FFs between 10.0% and 12.0%. The reported cases are shown as gray and black diamonds for the first and second sequencing of case A, respectively, and as a black square for case B. As observed in both cases, the NCV_y_:NCV_x_ ratio deviates significantly from that expected for male fetuses with FFs in the 5% and 10% ranges, respectively.

A 38-year-old patient (gravida 1) underwent assisted reproductive treatment at the end of December 2021, in which only one embryo was transferred. There were no previous abortions and no availability of preimplantation genetic testing for aneuploidy analysis before the transfer. At 11 weeks of gestation, the patient opted for cfDNA testing (NACE 24 test, Igenomix, Valencia, Spain) to screen for aneuploidies across the 23 pairs of chromosomes and to study copy number variations (deletions/duplications) larger than 7 Mb. The native analysis algorithm (Illumina VeriSeq NIPT Solution v2) classified the pregnancy as low risk for all conditions analyzed. The fetal sex was classified as male (XY) with an estimated native fetal fraction (FF_i_) of 5.0%; however, the Y-chromosome-based FF (FF_y_) was 2.0%. The normalized chromosome values for the sex chromosomes (NCV_x_ and NCV_y_) were 0.3 and 40.6. Given that these values were significantly lower than that expected for a male fetus with a 5.0% FF_i_ (Figure 1), the possibility of underlying clinical factors interfering with the result was discussed with the physician. The prescribing physician reported none of the previously described factors, such as altered known maternal karyotype, transfusions or transplants from a male donor, or the presence of a vanishing twin.

An ultrasound examination at 16 weeks of gestation revealed the presence of female genitalia in the fetus. To rule out a technical issue, cfDNA testing was repeated, yielding results consistent with the initial test (NCV_x_ = 0.2; NCV_y_ = 38.0; FF_i_ = 5.6%; FF_y_ = 1.9%).

To eliminate the possibility of a technical problem associated with the Illumina platform’s analysis algorithm, informed consent was obtained from the patient and the prescribing physician to re-analyze the data using an alternative bioinformatics algorithm based on that described by Bayindir et al., in 2015 (Bayindir et al., 2015). Paired-end sequencing data were aligned to the reference genome (*hg19*). Non-duplicated aligned reads were aggregated into 100-kb bins, adjusted for CG bias, and further aggregated into 5-Mb windows. Z-scores were calculated for autosomes, sex chromosomes, and 5-Mb bins. Sex was determined by verifying the presence of Y-chromosome-specific reads. Samples with ≥3 Y-specific bins containing >1 read were classified as male pregnancies. Two estimators were used for FF: one estimator based on the SeqFF method (Kim et al., 2015) and another estimator specific to the Y chromosome.

In the first and second sequencing, the Y chromosome Z-scores were 30 and 25, respectively, compatible with a male sex classification but still lower than expected for SeqFF-based estimated FFs (6.1% and 5.5%) [first seq: ZscoreX = −1.2; ZscoreY = 30.0; SeqFF = 6.1%; FF_y_ = 3.0%; second seq: ZscoreX = −0.7; ZscoreY = 25.4; SeqFF = 5.5%; FF_y_ = 2.7%].

**FIGURE 2:**
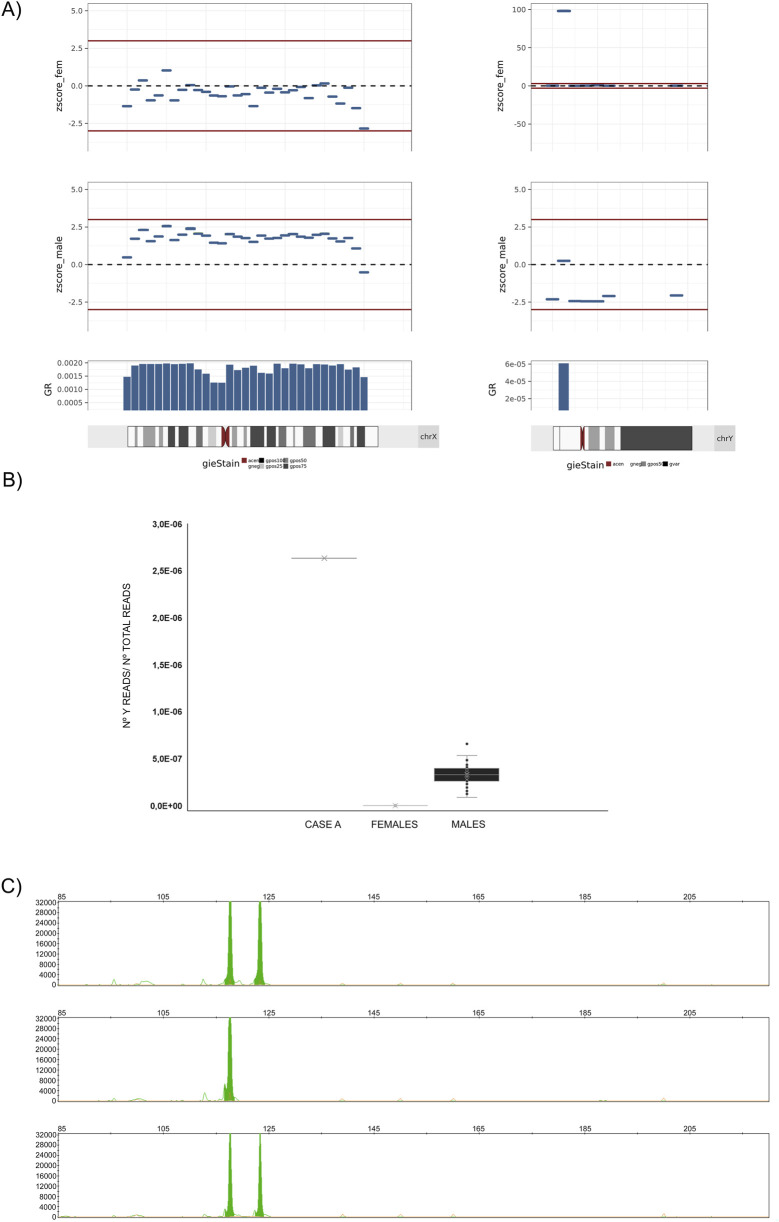
Analysis of the overrepresented genetic material belonging to the Y-chromosome. Case (A). **(A)** Left: Graphical plot of 5-Mb bin Z-scores across the X chromosome. Right: Graphical plot of Chr. Y 5-Mb z-scores across the Y chromosome. In all cases, the bin Z-score values have been calculated with regard to a female reference of normalcy (zscore_fem), or with regard to a male reference of normalcy (zscore_male). Between positions 5–10 Mb, a bin with a Z-score of approximately 100 is observed, a value 10 times higher than expected for a male fetus with a native fetal fraction (FF_i_) of around 5%. **(B)** Graphical representation of the ratio of Y chromosome reads to total reads within the genomic region of the Y chromosome spanning positions ChrY: 6,400,000–7400,000 (*GRCh37/hg19*) for (from left to right): CASE A, a set of 50 samples from pregnant patients with female fetuses (FFs between 5% and 12%), and a set of 50 samples from pregnant patients with male fetuses (FFs between 5% and 12%). In case A, the ratio exceeds by more than ten-fold the number of reads aligned to the Y chromosome in samples from patients with fetuses classified by the analysis algorithm as XY. **(C)** PCR amplification of the *amelogenin* (green peak) containing regions and fragment analysis of PCR products by capillary electrophoresis on AB3500 (ThermoFisher) and GeneMapper (rr). Top: patient; Middle: female reference; Bottom: male reference. The 119 bp peak represents *AMELX*, and the 124 bp peak represents *AMELY*. The marker under each allele peak represents the number of repeats.

An analysis of the distribution of DNA fragments along the Y chromosome revealed that DNA from this chromosome was not homogeneously present; Y-DNA fragments were concentrated solely to the cytogenetic region Yp11,2 covering the genomic coordinates ChrY:6,400,000_6,900,000 (*GRCh37/hg19*) (Figure 2A). A comparison with samples analyzed in the same set, including 50 fetuses classified as male and 50 as females without fetal sex discrepancies by ultrasound, revealed that fetuses classified by the native algorithm as female had no aligned DNA fragments in this genomic region. In contrast, fetuses classified as male exhibited a ratio of aligned Y-DNA fragments to total reads much lower than that found in the case described in this report (8 fold lower in the general male fetus sample set vs. case A) (Figure 2B).

The affected genomic location included the amelogenin gene *(AMELY*) and part of the Transducing-beta-like 1 (*TBL1Y)* gene (exons 1–6). To elucidate whether the overrepresentation of fragments was caused by an insertion of Y-chromosome genetic material into the maternal genome, fluorescent PCR was performed on white blood cell DNA using specifically designed primers. The amplification results revealed the presence of the expected female variant *AMELX* and the unexpected male variant *AMELY* in the maternal genome (Figure 2C).

### Patient 2 (case B)

A 41-year-old patient (gravida 1) became spontaneously pregnant at the end of January 2023. No previous abortions or other clinically relevant information were described in the medical history. At 14 weeks of gestation, the patient opted for extended cfDNA testing (NACE 24 test, Igenomix, Valencia, Spain). A low-risk result was obtained for all conditions evaluated, and the sex of the fetus was classified as male (NCV_x_ = −0.3; NCV_y_ = 40.7; FF_i_ = 10.8%; FF_y_ = 2.0%).

**FIGURE 3:**
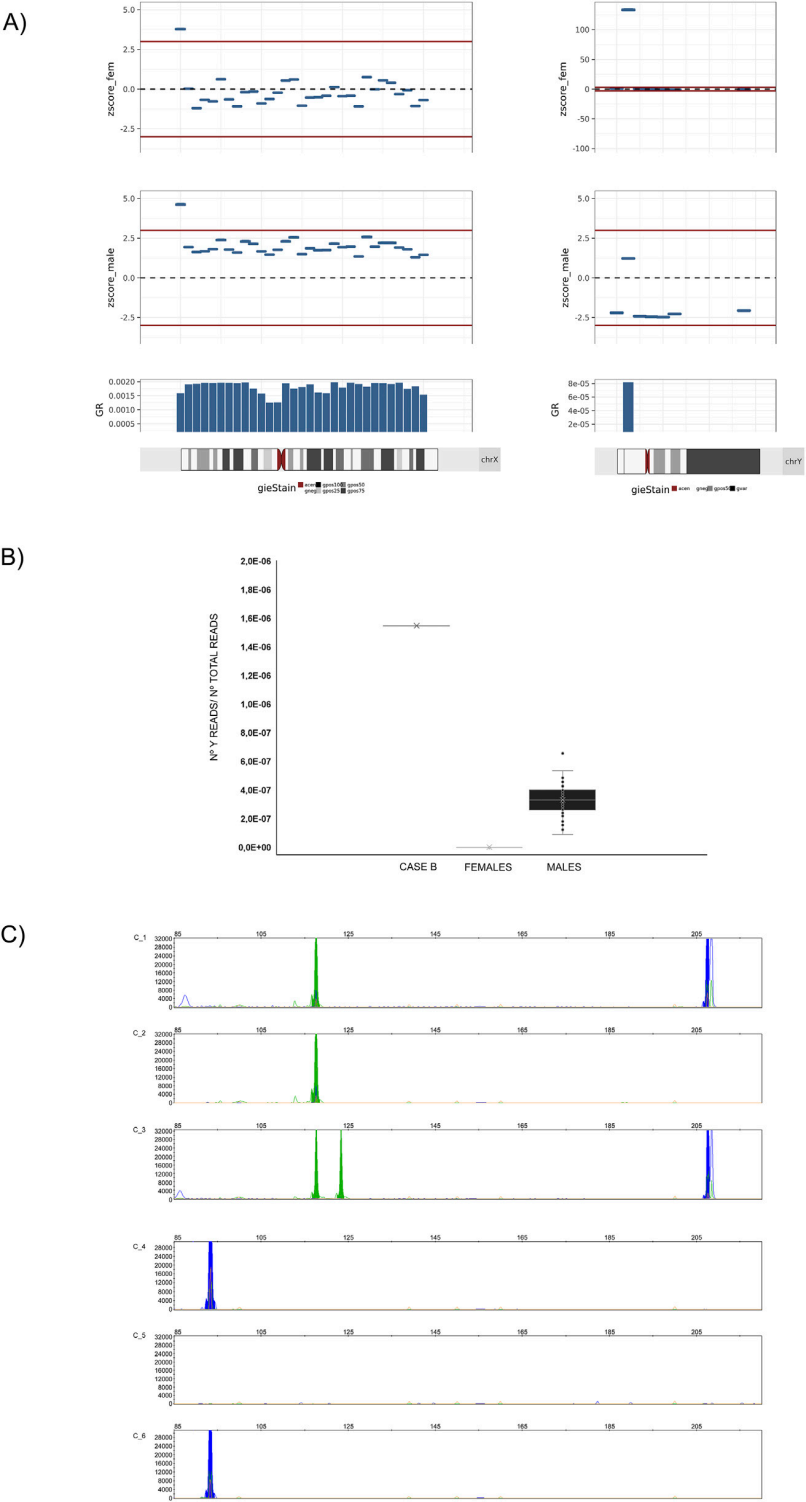
Analysis of the overrepresented genetic material belonging to the Y-chromosome. Case (B). **(A)** Left: Graphical plot of 5-Mb bin Z-scores across the X chromosome; right: Graphical plot of Chr. Y-5 Mb Z-scores across the Y chromosome. In all cases, the bin Z-score values have been calculated with regard to a female reference of normalcy (zscore_fem), or with regard to a male reference of normalcy (zscore_male). Between positions 5–10 Mb, a bin with a Z-score of approximately 125 is observed, a value 10 times higher than expected for a male fetus with a native fetal fraction (FF_i_) of around 11%. **(B)** Graphical representation of the ratio of Y chromosome reads to total reads within the genomic region of the Y chromosome spanning positions ChrY: 6,400,000–7400,000 (*GRCh37/hg19*) for (from left to right): CASE B, a set of 50 samples from pregnant patients with female fetuses (FFs between 5% and 12%), and a set of 50 samples from pregnant patients with male fetuses (FFs between 5% and 12%). In case B, the ratio exceeds by more than ten-fold the number of reads aligned to the Y chromosome in samples from patients with fetuses classified by the analysis algorithm as XY. **(C)** PCR amplification of *amelogenin* and *PRKY* and the Y:7,350,250–7,350,345 containing regions and fragment analysis of PCR products by capillary electrophoresis on AB3500 (ThermoFisher) and GeneMapper (rr): C_1 and C_4: patient; C_2 and C_5: female reference; C_3 and C_6: male reference. Green peak: The 119 bp peak represents *AMELX*, and the 124 bp peak represents *AMELY*. The blue peak at 208 bp represents *PRKY* and the blue peak at 96 bp is a region in chromosome Y at Y:7,350,241–7,350,341.

As it happened in Case A, an ultrasound examination at 16 weeks of gestation revealed the presence of female genitalia in the fetus. Based on our experience with case A, we obtained consent from the physician and the patient to conduct an exploratory analysis using the alternative bioinformatics pipeline without performing a second sequencing. This analysis aimed to evaluate the presence of aligned reads in specific regions of the Y-chromosome. The Z-score value for the Y chromosome was 40.02 with a SeqFF-based estimated FF of 8.8% and an FF_y_ of 3.5%; however, the Y-DNA fragment distribution analysis revealed alignment with the cytogenetic region Yp11.2, specifically between genomic coordinates ChrY: 7,000,000–7,500,000 (*GRCh37/hg19*) (Figure 3A). This region contains the pseudogene Protein Kinase Y-linked (*PRKY*), located in the Yp12 region. Similarly, the ratio of the number of Y-DNA readings to total readings in this region was significantly higher than that identified in a set of 50 samples classified as male fetuses (4.5 fold lower in the general male fetus sample set vs. case B) (Figure 3B).

After reporting the incidental findings to the prescribing physician and the patient, the decision was made to evaluate the patient for the previously described insertion. Fluorescent PCR on white blood cell DNA confirmed the insertion of the *PRKY*-including region into the patient’s genome (Figure 3C).

## Discussion

The prenatal diagnosis of sex discrepancies has historically relied on identifying ambiguous genitalia during ultrasound evaluations or observing genotype-phenotype discordance in cases involving preimplantation genetic diagnosis or invasive prenatal testing. The advent of cfDNA testing has significantly enhanced our ability to detect such discrepancies with greater precision. Estimates indicate that sex discrepancies occur in approximately 1 in every 1,500–2,000 pregnancies (Smet et al., 2020), a figure highlighted by the widespread adoption of cfDNA testing in modern prenatal screening.

We report a novel observation: sex discrepancies between cfDNA testing and ultrasound due to the insertion of Y-chromosome genetic material into the maternal genome. By analyzing cfDNA sequencing data and partitioning the Y-chromosome into 100-kb bins aggregated into 5-Mb windows, we identified a significant overrepresentation of reads aligned to the Yp11.2 cytoregion in two cases, exceeding typical male fetal levels by ten-fold.

In the first case, the insertion encompassed Y-chromosome material containing the *AMELY* gene and a portion of the coding region of the *TBL1Y* gene. *AMELY* codes for a matrix protein forming tooth enamel, constituting 90% of the total organic content (Richard et al., 2007). PCR products generated from *AMELX* and *AMELY* amplification can be discriminated using primers flanking a 6-base pair deletion in the gene’s first intron and differ between the two versions based on sex chromosomes. Sequence differences between *AMELX* and *AMELY* have been used to differentiate males from females with ambiguous phenotypes or to establish the gender in biological material for various purposes (Tozzo et al., 2013; Mannucci et al., 1994; Sullivan et al., 1993). In the second case, we described an insertion involving the *PRKY* gene, a member of the cAMP-dependent serine/threonine protein kinase gene family that shares homology with *PRKX* from Xp22.3 (Schiebel et al., 1997). *PRKY* and its X-chromosome homolog *PRKX* participate in various cellular processes, including signaling pathways implicated in cell growth and differentiation.

Notably, these insertions into the maternal genome are rare, with few documented cases in the scientific literature of females with a 46, XX karyotype harboring such Y-chromosome gene insertions. Among the few reported cases, Stapleton et al. (2008) identified a family in which three females carried both the male and female variants of the *AMELY* gene, suggesting Mendelian inheritance and highlighting the problem of using only *AMELY* as a sex-specific marker (Stapleton et al., 2008). Cases of *AMELY*-negative males are more frequently detected worldwide; the genetic mechanisms underlying *AMELY* dropout involve deletions of different sizes, encompassing the *AMELY* locus, and mutations in the primer-binding region of the *AMELY* allele (Jobling et al., 2007; Butler, 2011). Deletions in the Yp11.2 region, a major cause of the *AMELY* null allele, are often combined with the absence of adjacent Y-STR loci *DYS456* and/or *DYS458* (Jobling et al., 2007; Cadenas et al., 2007; Ma et al., 2012; Chen et al., 2014; Chang et al., 2007; Ou et al., 2012; Kumagai et al., 2008; Turrina et al., 2009; Borovko et al., 2015).

The significance of these findings lies not only in the phenotypic and clinical implications for carrier patients but also in the potential to enhance fetal sex prediction in current bioinformatic analysis pipelines for cfDNA testing. The evolution of bioinformatic pipelines has led to remarkable improvements in the sensitivity and specificity of cfDNA testing for fetal sex determination and aneuploidy detection. Early PCR-based methods displayed sensitivities and specificities in the range of 95%–98% (Lo et al., 1998). The advent of massively parallel sequencing and advanced bioinformatics have improved these metrics to over 99% for fetal sex determination (Kotsopoulou et al., 2015).

Several methods have been proposed to predict fetal aneuploidies, typically involving genome binning, normalization techniques, and Z-score calculations to evaluate variation in the normalized fragment count in each bin. Comparisons are made with euploid reference samples (e.g., cn. MOPS (Talevich et al., 2016) and CNVkit (Klambauer et al., 2012), between sample variations (e.g., Wisecondor (Straver et al., 2014) and WisecondorX (Raman et al., 2019), or through reference-free approaches (e.g., FREEC (Boeva et al., 2011), QDNAseq (Scheinin et al., 2014), and BIC-seq2 (Xi et al., 2016). Although these tools facilitate cfDNA testing analysis, they remain challenging to implement in a clinical setting in many laboratories worldwide due to intensive data processing and the need for advanced bioinformatics skills. Additionally, the diversity of technologies and algorithms employed can prompt significant variability in results, and we lack a comprehensive quantification of this variation (Duboc et al., 2022). Because of the inherent differences among methodologies, clinical laboratories should leverage the bioinformatics available to help navigate complex cases with clinicians.

Therefore, we must develop a protocol for cases with an anticipated sex discrepancy, even though reaching a consensus on the best algorithm or platform for maximum sensitivity and specificity remains challenging. For prenatally reported fetal sex discrepancies despite ultrasound examination by an experienced examiner after 16 weeks of gestation, a complete history and physical examination always represent the initial steps to guide further evaluation. In parallel, we must rule out human error by confirming correct sample labeling.

We recommend conducting a detailed review of existing data before repeating sequencing on an independent sample to rule out potential sample errors. This review should focus on identifying overrepresented Y chromosome regions compared to that expected for a male fetus. In the case of identifying such overrepresentation, the prescribing physician must be informed, and the maternal genome should be investigated for possible insertions of Y-chromosome genetic material before performing any unnecessary invasive procedures.

We acknowledge there are some important limitations for the proposed protocol. The occurrences described in this article are rare, making generalizing findings to broader populations challenging. The advanced bioinformatics methods employed may not be readily accessible in all clinical settings, potentially limiting widespread adoption of the proposed protocols. And the reliance on cfDNA testing and bioinformatics could lead to misinterpretations of results if not carefully managed, particularly in the absence of confirmatory tests. Thus, major platforms offering cfDNA testing should incorporate these considerations into their decision algorithms. Based on the evidence presented here, the presence of isolated bins on the Y chromosome with Z-scores elevated to levels not expected in pregnancies bearing a male fetus should be carefully analyzed when making a final sex calling. This approach would prevent undesirable situations and alleviate the anxiety of the expectant mother concerning potential clinical issues for her or the fetus.

If none of the aforementioned conditions are confirmed, the physician should discuss the case within the context of a multidisciplinary team. This team should be aware of all developments and findings to prepare a comprehensive care pathway for the family. Based on this, a protocol of action could be defined, including a series of tests such as 1) direct assessment of the fetal sex chromosome distribution (ploidy, copy number variations, or mosaicism) through amniocentesis accompanied by a genetic counseling interview; 2) further investigation for Y-chromosome-specific material using an *SRY* probe which may help in evaluating sex chromosome anomalies, including translocation to the X chromosome or autosomes in XX individuals or mosaicism; and/or 3) analysis for single-gene conditions causing disorders of sexual differentiations, such as congenital adrenal hyperplasia or androgen insensitivity and a wide range of genetic syndromes that impact external genitalia.

## Conclusion

These findings underscore the complexity of prenatal sex determination and the potential of genetic anomalies in influencing cfDNA testing results. By incorporating detailed bioinformatic reviews and considering maternal genomic contributions, we can improve the accuracy of fetal sex prediction and minimize unnecessary invasive procedures, ultimately enhancing prenatal care and reducing anxiety for expectant mothers.

## Data Availability

The raw data supporting the conclusions of this article will be made available by the authors, without undue reservation.
